# Core Competencies to Promote Consistency and Standardization of Best Practices for Digital Peer Support: Focus Group Study

**DOI:** 10.2196/30221

**Published:** 2021-12-16

**Authors:** Caroline Collins-Pisano, Juan Velez Court, Michael Johnson, George Mois, Jessica Brooks, Amanda Myers, Anjana Muralidharan, Marianne Storm, Maggie Wright, Nancy Berger, Ann Kasper, Anthony Fox, Sandi MacDonald, Sarah Schultze, Karen Fortuna

**Affiliations:** 1 Department of Psychiatry Dartmouth College Hanover, NH United States; 2 National Latino Behavioral Health Association Cochiti Lake, NM United States; 3 The Commission on Accreditation of Rehabilitation Facilities International Tucson, AZ United States; 4 School of Social Work University of Georgia Athens, GA United States; 5 Department of Psychiatry School of Medicine and Public Health University of Wisconsin–Madison Madison, WI United States; 6 The Heller School for Social Policy and Management Brandeis University Waltham, MA United States; 7 Department of Psychiatry University of Maryland School of Medicine Baltimore, MD United States; 8 Department of Public Health University of Stavanger Stavanger Norway; 9 Families in Trauma and Recovery Fife in Scotland United Kingdom; 10 University of Massachusetts Lowell Lowell, MA United States; 11 Kasper Connects Portland, OR United States; 12 Tennessee Mental Health Consumer's Association Nashville, TN United States; 13 International Association of Pre-Menstrual Disorders Boston, MA United States; 14 School of Social Work University of New Hampshire Durham, NH United States; 15 Department of Psychiatry Geisel School of Medicine Dartmouth College Hanover, NH United States

**Keywords:** COVID-19, peer support, competencies, training, digital

## Abstract

**Background:**

As digital peer support is quickly expanding across the globe in the wake of the COVID-19 pandemic, standardization in the training and delivery of digital peer support can advance the professionalism of this field. While telehealth competencies exist for other fields of mental health practice, such as social work, psychiatry, and psychology, limited research has been done to develop and promote digital peer support competencies.

**Objective:**

The goal of this study is to introduce the coproduction of core competencies that can guide digital peer support.

**Methods:**

Peer support specialists were recruited through an international listserv and participated in a 1-hour virtual focus group. A total of four focus groups were conducted with 59 peer support specialists from 11 US states and three countries.

**Results:**

Analysis was conducted using the rigorous and accelerated data reduction (RADaR) technique, and 10 themes were identified: (1) protecting the rights of service users, (2) technical knowledge and skills in the practice of digital peer support, (3) available technologies, (4) equity of access, (5) digital communication skills, (6) performance-based training, (7) self-care, (8) monitoring digital peer support and addressing digital crisis, (9) peer support competencies, and (10) health literacy (emerging). The authors present recommendations based on these themes.

**Conclusions:**

The introduction of digital peer support core competencies is an initial first step to promote the standardization of best practices in digital peer support. The established competencies can potentially act as a guide for training and skill development to be integrated into US state peer support specialist competencies and to enhance competencies endorsed by the Substance Abuse and Mental Health Services Administration (SAMHSA).

## Introduction

In the wake of the COVID-19 pandemic, digital peer support has rapidly expanded across the globe. Digital peer support is live or automated peer support services delivered through technology media, such as peer-to-peer networks on social media, peer-delivered interventions supported with smartphone apps, and asynchronous and synchronous technologies [1]. Peer support has been described as social emotional support, frequently coupled with instrumental support [2]. It is mutually offered or provided by persons with a mental health condition or substance use disorder to others sharing a similar mental health condition or substance use disorder. Peer support has augmented mental and physical health care by providing support between clinical encounters [3]. Peer support specialists enhance mental health service users’ hope, quality of life, social support, and recovery; are linked to reduced symptoms among mental health service users; and help to improve engagement in mental and physical health services [4,5].

Peer support is defined as an essential mental health service by the World Health Organization [6]. As referenced in the scientific evidence, peer support has expanded from in-person services to telemental health services prior to the COVID-19 pandemic (eg, warmlines) [7]. Prior to the COVID-19 pandemic, there was quite a widespread existence of digital peer support programming. For example, in a study led by Rotondi and colleagues [8], peers used a website and home computers to deliver psychoeducational therapy to individuals with schizophrenia and their family and friends. Participants in that study reported statistically significant improvements in psychiatric symptoms, including fewer positive symptoms. Digital peer support has also been offered through many different platforms, such as Facebook, smartphone apps, and fitness trackers, to promote fitness and self-monitored exercise [9,10]. However, due to the COVID-19 crisis and related lockdown and social distancing measures, digital peer support has rapidly expanded and includes various technology modalities. For example, digital peer support, once commonly offered through the telephone, has transitioned to be offered through technologies such as virtual reality and video games [11]. Peer support specialists are developing and coproducing digital peer support technologies with peer and nonpeer scientists (ie, peer support specialists are offering their lived experience expertise to partner in the development of digital peer support).

Peer-led, medical self-management programs show promise for significantly improving mental health service user activation (ie, “patient activation” is defined as patients’ willingness and efficacy in managing their own health and recovery) and the ability of the individual to manage their illness and health behaviors, collaborate with providers, maintain function, and prevent health decline [12]. Craig et al [13] investigated the feasibility of employing mental health service users as health care assistants within an assertive outreach team. The assertive outreach team consisted of an associate specialist psychiatrist, eight case managers with a nursing background, and a psychiatrist consultant. The team provided service to persons with a record of poor engagement in care, problematic behavior, substance abuse, and multiple hospitalizations. A total of 45 clients of the assertive outreach team were randomly assigned to either the standard team or the assertive outreach team plus a mental health service user who acted as a health care assistant. Craig et al [13] found that individuals with a lived experience of a mental health challenge were valued as role models who had “insight” into the care system and the resource capacity to advocate for clients within the clinical team. In addition, Clarke et al [14] found that an Assertive Community Treatment (ACT) team with case managers had a lower likelihood of psychiatric hospitalization than a nonconsumer-staffed ACT team. Clarke et al [14] theorized that mental health service users who had been hospitalized themselves may have been more motivated to keep service users out of the hospital and may have had greater tolerance for psychotic behavior or extreme states. Some studies have found peer-assisted care is not always more effective than standard or clinical care [15]. However, peer support significantly increases self-reported social contacts with service users and professional staff and enhances social networks [15].

As digital peer support quickly expands across the globe, development of digital peer support competencies is needed to develop a framework for consistency and standardization of best practices. Formal academic training programs for other mental health professionals, such as social workers, psychiatrists, psychologists, medical professionals, and community health workers, address best practices for implementing telemental health services [16]. While core competencies have been developed for in-person peer support (ie, Core Competencies for Peer Workers developed by the Substance Abuse and Mental Health Services Administration [SAMHSA]), to date, digital peer support competencies have not been considered [17]. In 2015, SAMHSA published core competencies for peer support specialists in an effort to identify the skills and abilities needed to provide support to people recovering from a mental health or substance use condition [17]. For example, SAMHSA identified the ability to engage peers in collaborative relationships and value communication as important competencies for in-person peer support specialists. Peer support’s rapid transition to digital peer support during the COVID-19 pandemic requires the *expansion* and *redefinition* of skills and abilities for peer support specialists who are now offering services and support online through digital platforms.

Digital peer support has the potential to expand the reach and practices of in-person peer support. Promising evidence indicates that digital peer support specialists may enhance service users’ ability to live in community settings [18], improve the impact of peer support without the need of in-person sessions [19], and promote engagement in mental and physical health services [18]. Digital peer support sessions have no geographical or time limitations, engage service users in digital mental health, and may increase access to mental health care for hard-to-reach groups, such as rural or home-bound service users.

Introducing core competencies is the first step to standardizing telehealth practices for this mental health workforce. Core competencies could potentially facilitate consistency among digital peer support training programs across state and international borders. The current mental health landscape employs multiple models of peer support practice [20], making consistency across states difficult to ascertain. Similar to other fields, core competencies have the ability to promote best practices, guide delivery, inform training programs, assist in developing standards for certification in digital peer support, and assist in the evaluation of work performance. As such, the purpose of this study was to inform the development of core competencies for digital peer support specialists as an initial first step to promote the consistency and standardization of best practices in digital peer support.

## Methods

Using a convenience sample, peer support specialists that currently offer digital peer support services were recruited by an international listserv that includes 1500 peer support specialists. Focus groups were determined as the most appropriate data collection methodology to ensure that the perspectives of peer support specialists were included in the initial development of competencies. Participants were eligible if they were older than 18 years of age and were a peer support specialist. Participants were asked to complete an online presurvey with questions on demographic information (eg, age, race, and gender) to ensure variation in focus group participants, and they participated in a 1-hour online focus group. The questions in the focus group interview guide were coproduced with four peer and nonpeer academic scientists and four peer support specialists using the Peer and Academic Partnership to determine what essential knowledge and abilities are necessary for effectively delivering digital peer support [21]. The Peer and Academic Partnership is the collaboration of academic researchers and certified peer support specialists to guide the development of interventions, trainings, and academic materials [21]. The Peer and Academic Partnership has been used to develop mobile apps, shared decision-making tools, and instruments to measure community-engaged research [21].

Interview guide questions included the following: “What essential knowledge does a peer support specialist need to offer digital peer support?” “What are the essential abilities peer support specialists need to offer digital peer support?” “How do these essential skills vary by lived experience (eg, mental health, physical health, substance use challenges, veteran status, aging, and racial or ethnic diversity)?” and “How do peer support practice standards as defined by the Substance Abuse and Mental Health Services Administration (SAMHSA) align or not align with what we have spoken about today?” SAMHSA’s Core Competencies for Peer Workers includes the following: (1) engages peers in collaborative and caring relationships; (2) provides support; (3) shares lived experiences of recovery; (4) personalizes peer support; (5) supports recovery planning; (6) links to resources, services, and support; (7) provides information about skills related to health, wellness, and recovery; (8) helps peers to manage crises; (9) values communication; (10) supports collaboration and teamwork; (11) promotes leadership and advocacy; and (12) promotes growth and development [17].

The development of competencies is not achieved through individual processes, but rather through collaborative group processes in social contexts [22]. For example, in excluding older adults and their unique perspective of living in a nursing home, we are not able to learn about the person in their specific environment. To reproduce such a group process and to build off of other opinions, we aligned our data collection to promote cross-individual opinions. As such, we employed a series of focus groups to develop the digital peer support competencies. The final set of competencies based on focus group findings incorporated member-checking to ensure accuracy and face validity. Member-checking was used to enhance the credibility of data analysis and participant involvement [23]. Researchers presented data transcripts to all participants for comment, and participants were asked to review the transcripts to determine if the words match their intended meanings and to validate the researcher’s interpretation of the data [23].

The analysis of digital peer support competencies, within the framework of peer-run organizations and Medicaid-reimbursable organizations, was based on four 1-hour focus group discussions with a total of 59 participants across the four focus groups, which took place in October 2020. The focus groups were carried out by two authors (CCP and A Myers) using the interview guide. The interview guide was successfully tested in a pretest. Focus group participants were emailed the interview guide 24 hours prior to the focus group to prepare for the actual focus group. Focus group facilitators had 1 to 5 years of experience conducting focus groups with the sample of interest. To facilitate participation, focus group facilitators developed a set of ground rules with participants, including (1) the confidentiality of the focus group, (2) allowing everyone time to speak, (3) having mutual respect for one another, and (4) being available for follow-up. Focus group facilitators asked every participant for their input on each interview question.

The focus group discussions were recorded digitally, transcribed, and anonymized. Focus groups were conducted until there was saturation (ie, when sampling more data would not lead to more information) [23]. The data were analyzed using the rigorous and accelerated data reduction (RADaR) technique, a team-based approach to coding and analyzing qualitative data [24]. The RADaR method helps to streamline the process of qualitative data analysis and produce qualitative results quickly and thoroughly through its ability to organize, reduce, and analyze data in user-friendly software packages, such as Microsoft Excel [24]. Aligned with the RADaR methodology, data transcripts were formatted into an all-inclusive Excel spreadsheet. The Excel spreadsheet included column headings such as question, participant number, and response. Team members worked in groups to assign codes to each response. After the all-inclusive Excel spreadsheet was produced, the data table was reduced to include only content relevant to the interview questions. The remaining text and codes were then organized into themes. Common codes were derived from the focus groups by carefully reviewing the transcribed text and were grouped together and organized under overarching themes. Consistent with the RADaR methodology, themes were determined by the incidence at which a code aligned with an overarching theme (see Results). To ensure the text and codes were interpreted correctly and were correctly organized into themes, the process of member-checking was employed. Member-checking is a qualitative method used to validate findings, resolve conflicting results, and assess the trustworthiness of qualitative results [25]. The percentage for each theme was found by dividing the frequency in which the theme was present in the focus group quotes by the total number of focus group quotes.

The Committee for the Protection of Human Subjects at the Dartmouth-Hitchcock Institutional Review Board approved the project.

## Results

### Participants

A total of 59 peer support specialists participated in one of four focus groups. Over 78% of the participants had a degree-level education, and the majority of participants were female (Table 1). The participants were from 11 states and three countries: the United States, Canada, and Australia.

We identified 76 codes and a set of 10 themes relating to the development of digital peer support competencies. Themes covered different knowledge and skills that the participants believed were integral to the practice of digital peer support. The 10 themes were as follows: (1) protecting the rights of mental health service users, (2) technical knowledge and skills in the practice of digital peer support, (3) available technologies, (4) equity of access, (5) digital communication skills, (6) performance-based training, (7) self-care, (8) monitoring digital peer support and addressing digital crisis, (9) peer support competencies, and (10) health literacy (emerging) [26] (Table 2).

**Table 1:** Participant characteristics.

Sociodemographic characteristics		Participants (N=59), n (%)
**Gender**		
	Male	11 (19)
	Female	35 (59)
	Missing	13 (22)
**Age (years)**		
	19-26	3 (5)
	27-49	15 (25)
	50-64	23 (39)
	≥65	5 (8)
	Missing	13 (22)
**Racial background**		
	White	35 (59)
	Black or African American	7 (12)
	Asian	1 (2)
	More than one race	3 (5)
	Missing	13 (22)
**Highest grade in school completed**		
	Completed high school or GED (General Educational Development)	3 (5)
	Some college	10 (17)
	Completed college or technical school	4 (7)
	Completed associate degree	11 (19)
	Completed bachelor’s degree	9 (15)
	Some graduate school	2 (3)
	Completed master’s degree	7 (12)
	Missing	13 (22)
**Current workplace organization type**		
	Peer run	12 (20)
	Medicaid reimbursable	7 (12)
	Commercial health system	4 (7)
	Other	23 (39)
	Missing	13 (22)
**Employment status**		
	Full time	32 (54)
	Part time	6 (10)
	Volunteer	1 (2)
	Unemployed	4 (7)
	Student	2 (3)
	Missing	14 (24)
**Type of lived experience impacting participant**		
	Bipolar disorder	8 (14)
	Major depression	13 (22)
	Other mental health concerns	6 (10)
	Alcohol use disorder	3 (5)
	Opioid use disorder	3 (5)
	Other substance misuse concerns	4 (7)
	Obesity	1 (2)
	Heart disease	1 (2)
	High blood pressure	1 (2)
	Diabetes	1 (2)
	Other physical health conditions	4 (7)
	Missing	14 (24)
**Offers digital peer support**		
	Yes	35 (59)
	No	10 (17)
	Missing	14 (24)
**If offering digital peer support, modes by which digital peer support is provided**		
	Smartphone app	15 (25)
	Text messaging	21 (36)
	Phone calls	25 (42)
	Videoconference	24 (41)
	Social media	5 (8)
	Virtual reality	2 (3)
	Other	2 (3)
**Training received to offer digital peer support**		
	Digital peer support certification (short course)	22 (37)
	Learned by doing	9 (15)
	Have not received formal training	10 (17)
	Other	4 (7)
	Missing	14 (24)
**Credentials**		
	Digital peer support certification (short course)	22 (37)
	Recovery coach training	8 (14)
	Certified peer support specialist	34 (58)
	Noncertified peer support specialist	5 (8)
	Forensic peer support	5 (8)
	Certified older adult peer support specialist	2 (3)
	Wellness Recovery Action Plan	17 (29)
	Whole Health Action Management	6 (10)
	Emotional CPR	4 (7)
	PeerTECH	2 (3)
	Digital peer support engineer	1 (2)
	None of the above	2 (3)
	Other	6 (10)
**Types of digital peer support technologies developed by participant**		
	Filmed digital peer support videos	5 (8)
	Developed scripted text messages	8 (14)
	Developed scripted social media posts	3 (5)
	Developed digital peer support program content	13 (22)
	Developed smartphone apps	3 (5)
	Developed videoconference software	1 (2)
	Other	9 (15)

**Table 2:** Digital peer support core competencies.

Theme	Description	Observable behavior of competencies suggested by peer support specialists	SAMHSA^a^ competencies
Protecting the rights of service users	Understanding of cybersecurity and how to protect the information and the privacy of service users Understanding how to protect the confidentiality of service users Complying with local, state, national, and international (if applicable) privacy laws and regulations	Evaluating how data are being collected on certain digital platforms Obtaining informed consent from service users Facilitating meetings with service users in settings where confidentiality can be maintained Understanding the Health Insurance Portability and Accountability Act (HIPAA) privacy laws and whether devices are HIPAA compliant	N/A^b^
Technical knowledge and skills in the practice of digital peer support	Acquiring the skills and knowledge necessary to comfortably use technology devices and platforms	Ability to understand different platforms and devices and teach service users how to comfortably use their devices and digital platforms that are required for their digital peer support, both in person and remotely	N/A
Available technologies	Ability to provide information and support through devices and platforms accessible to, and preferred by, service users	Adapting to the digital preference of the service user; for example, with young adults communicating via text	Supports recovery planning
Equity of access	Ability to connect with service users from diverse backgrounds and locations Knowledge of resources around technology accessibility	Acquiring knowledge of tools and technological resources available for special populations; for example, knowledge on accessibility of resources for rural populations	N/A
Digital communication skills	Skills that digital peer support specialists need to effectively communicate via digital platforms	Empathetic listening and constant “checking-in” with the service user when body language is less visible	Values communication Shares lived experiences of recovery Links to resources, services, and supports Provides information about skills related to health, wellness, and recovery
Performance-based training	Standardized training in which peer specialists acquire the knowledge and skills of digital support; necessary to peer support specialists’ ability to transition to digital peer support	Practice phone call or virtual interaction between supervisor and digital peer support specialist to assess knowledge and skills	Engages peers in collaborative and caring relationships Provides support Promotes growth and development Supports collaboration and teamwork
Monitoring digital peer support and addressing digital crisis	Aware of agency’s crisis plan and of resources available to support service users between meetings	Transparency with service users around digital peer support specialist’s role and training around digital crisis intervention	Helps peers to manage crises
Peer support competencies	Maintaining the core competencies of peer support within the practice of digital peer support	Ability to cultivate hope, empathy, engagement, and mutuality and to share lived experiences	All of the SAMHSA competencies
Self-care	Skills needed to ensure digital peer support specialists are caring for themselves as well as they care for others Ability to set boundaries, limit digital fatigue, and monitor mental exhaustion	Planning-in breaks throughout the day for self-care; for example, physical activity Discussing boundaries with service users and setting a plan for when the peer support specialist is and is not available during the day	N/A
Health literacy	Knowledge of digital health literacy; digital health literacy is the “ability to seek, find, understand, and appraise health information from electronic sources and apply the knowledge gained to addressing or solving a health problem” [26]	Creating a checklist around basic digital health literacy	N/A

^a^SAMHSA: Substance Abuse and Mental Health Services Administration.

^b^N/A: not applicable; this theme had no related SAMHSA competencies.

### Protecting the Rights of Service Users

The most prevalent theme was protecting the rights of service users. This core theme was made up of four key subthemes: confidentiality, security, privacy, and informed consent. Protecting the rights of service users constituted 19.2% (28/146) of the themes discussed in the focus groups. Peer support specialists questioned how telehealth policy and regulation would inform the use of different technologies and platforms. A peer support specialist mentioned, “telehealth rules are going to affect what you can and can’t do.” Multiple participants cited understanding and evaluating the security features of technology as essential knowledge for digital peer support. For example, one participant said, “one piece of essential knowledge is how to evaluate the security and the privacy aspects of a digital platform.” Guidelines for confidentiality protections were frequently encouraged by participants. Participants recommended constructing agreements with service users on privacy and the protection of personal information. A participant reported that there should be “guidelines that people have to agree to before they undertake support so that they know...they can’t have people around that are going to be hearing stuff that might be confidential.” Participants also referred to the importance of informed consent. Transparency about the digital peer support role and regarding shared information was recommended. For example, a participant stated, “it’s about transparency and trust...If their information is being shared or if I can see their phone number, I’m going to be honest...we have to be up-front.”

### Technical Knowledge and Skills in the Practice of Digital Peer Support

The second theme, technical knowledge and skills in the practice of digital peer support, constituted 15.1% (22/146) of the themes discussed in the focus groups. Participants recommended that digital peer support specialists should have the knowledge necessary to comfortably use technology in a meeting. A participant stated, “so when I’m doing one-on-one peer support over the phone or on a Skype call, I need to know how to use the technology.” The majority of participants believed that digital peer support specialists should also have the basic skills to use different platforms and devices. For example, one participant stated, “they need to understand the difference in how to use the different platforms.” The ability to teach service users how to access and comfortably use different digital platforms, devices, and resources was also encouraged. A participant stated the following:

[Digital peer support specialists] need some basic knowledge of computers and a virtual format, like accessing applications like Zoom, not only to be able to navigate it, but to teach others how to access it and to navigate it on different platforms, like a computer and a mobile phone.

However, peer support specialists also encouraged digital peer support specialists to use a blend of both digital and nondigital resources. For example, a participant stated, “I mail out the material before the groups start so any time I get a new participant, I make sure that they have the material instead of just doing the screen sharing.” Multiple peers support specialists also emphasized the skill of knowing when to shift to different platforms or virtual exercises when interacting with service users. A participant stated, “I think being able to pay enough attention to know when the person on the other end is losing interest and knowing, ‘okay, maybe it’s time to switch gears.’” Lastly, multiple participants also referred to the importance of understanding technical troubleshooting. For example, a participant stated, “the ability to handle technical difficulties if they arise, and not panic” was essential for digital peer support specialists.

### Available Technologies

The third theme, available technologies, included the subtheme of preference. The theme of available technologies constituted 11.6% (17/146) of the themes discussed in the focus groups. Many peers emphasized how the preferred type of device or digital platform may vary by mental health condition and age group. The type of mental health condition may affect whether or not the service user wants to communicate with or without a webcam and visual component. A participant stated the following:

I know for just living with substance use disorder, sometimes, the idea of a video chat can be very triggering for some because during certain drug uses, especially in the heavy forms of it, it’s actually used as a platform to connect with other people who are using the same drug as you as a means to sort of show off and that was, I know when Zoom came around for therapeutic services, there were a lot of people in the substance use community that are in recovery that I’ve spoken with that have definitely said that it’s very hard for them to be on any kind of webcamming.

Many peer support specialists also suggested technology preference may differ by age group. Based on experience, younger adults seemed to prefer communicating via text, while older adults preferred communicating via tablet; for example, a participant stated, “many of the young adults that I work with in our first episode psychosis program don’t have a tolerance or a desire to sit through a 1-hour video call like we’re doing now. They prefer texting.” Another added, “with older adults...a tablet is the technology that is most preferable because of the size of it.”

### Equity of Access

The fourth theme was equity of access and included the subthemes accessing technology, accessibility of platforms for people with physical challenges, accessibility of platforms for people with environmental challenges, and cultural competence. The theme of equity of access constituted 11.0% (16/146) of the themes discussed in the focus groups. The ability to connect with service users from different backgrounds and cultures was cited as an essential ability for digital peer support specialists. One participant mentioned the following:

Well, I think that engagement is a cultural matter of having cultural humility and understanding and empathy for a person’s worldview. And being able to meet a person where they are whether it’s in a rural situation, whether it’s in a homeless situation, whether it’s in an urban high rise, you know, there needs to be a way that the specialist can extend him or herself, to make an effort to understand the environment out of which a person served is coming and where they’re at.

Participants encouraged digital peer support specialists to gain knowledge of technological resources for special populations, like rural service users, who may have difficulties accessing technology. For example, a participant stated the following:

Having tools to be able to be proactive and kind of have insight and awareness to meet the needs of various populations in terms of location and access and or maybe having, for example...knowledge of different programs that may offer certain phones, and that kind of awareness to meet the needs of different populations as well.

Knowledge of platforms and devices that are accessible to people with physical challenges was also encouraged:

There are like, automated, like voice-to-text generators that you can get that will provide subtitles for people that have sensory issues. And that’s something that can make it accessible for people who might not otherwise be able to attend. So looking at those sorts of accessibility options is something that’s probably important or asking people who are going to be attending whether they have any accessibility needs and trying to work out a way to accommodate them.

Many participants also encourage the continuation of digital peer support because of its ability to reach marginalized populations. A participant stated, “we need to maintain this online stuff because it is accessible to so many people that would probably not be accessing anything otherwise.”

### Digital Communication Skills

The fifth theme, digital communication skills, constituted 10.3% (15/146) of the themes discussed in the focus groups. The majority of participants cited effective communication as a necessary component to digital peer support. With the lack of body language and, at times, visual interaction, multiple peer support specialists recommended that digital peer support specialists rely on verbal cues and engagement skills while interacting with service users virtually:

Part of the biggest thing for us as peers is to listen...So how do you do that without being able to convey verbal cues?...I just have to use my verbal cues, and then just kind of, you know, let him know, I’m there. I’m with you. I understand, I hear you.

Participants cited strong group facilitation skills as a necessity for digital communication. For example, a participant stated, “it’s really important to have good facilitation skills, especially if you’re doing groups online, because it can be harder than in person because people don’t always see the cues, they don’t always notice the body language of other people.” Comfort with digital platforms and devices was believed to facilitate digital communication skills:

I think a peer support specialist needs to have a comfort with whatever virtual platform they are using to interact with the service user. And beyond that, just needs to really extend him or herself in kind of a wholehearted way, to convey their concern, to convey hope, to convey their experience to the service user.

Overall, peer support specialists suggested that, as with in-person peer support, digital peer support should focus on the building of relationships, no matter what digital device is being used. For example, one participant said, “very simply, engagement is about building relationship. If you don’t build a relationship you cannot engage. So it doesn’t matter if we’re talking smartphones, tablets, laptops, Webex, Zoom. It’s about building a relationship.”

### Performance-Based Training

The sixth theme, performance-based training, constituted 8.9% (13/146) of the themes discussed in the focus groups. Many participants encouraged digital peer support specialists to attend a digital peer support course. A participant stated, “I think the digital peer support course would be really helpful.” Other peer support specialists believed there should be agency or state-specific trainings for digital peer support specialists:

In addition, it would be really cool if there was another layer of training. So for a specific agency, for example, if there were certain regulations that were different...or [if there were] certain requirements or different kind of policies at the state level.

Some participants recommended agencies or supervisors give feedback to peers based on their digital peer support skills. One participant believed “there should be a competency quiz so maybe a quiz or checking back and having them be able to repeat that information.” Practice digital conversations were also recommended as a training option for digital peer support specialists:

After completing the training what we do is we have a fake call or fake message conversation where for an hour the supervisor pretends to be in crisis and reaches out and we have to provide ample support to them and then they critique us on everything that we said after the hour’s up and they tell us if we are allowed to go on and do peer support provider.

### Self-Care

The seventh theme, self-care, was an emerging theme that constituted 8.9% (13/146) of the themes discussed in the focus groups. Many participants recommended planning in times throughout the day for self-care:

Scheduled blocks throughout the day that you’re not in a meeting so that 1) you can avoid fatigue and 2) get other things done...That’s part of the boundary of self-care that we practice, putting in those blocks of time so I don’t have a meeting during that time because I need to go for a walk, or I need to finish my emails or a report or whatever it might be.

Some peer support specialists encouraged physical activity as a break from digital peer support. For example, “getting up for a walk” or “physical activities have helped a lot with my own personal fatigue.” Participants also categorized the ability to ask for help as an important aspect of self-care:

I think an essential ability is to that when you are having those tough moments to make sure that people reach out for help, so that they don’t feel like they’re struggling on their own, and that they do get feedback on some of those maybe tougher cases...Because I think with peer support, you know, we talk about the trauma informed, because it’s a part of our lived experience to share those pieces where it builds connection, we might get retraumatized all over again.

Many participants referenced the ability to separate work from personal life and the ability to set boundaries as essential skills when providing digital peer support. A participant stated, “having that maintenance of boundaries so you don’t wear yourself out.” Peer support specialists recommended the formation of plans and set time periods for when service users could and could not call. For example, one participant shared, “come up with a plan to be able to have set time periods that you can call, whether it be certain hours that you know that person’s not going to be there to strictly making it within those hours...just setting those agreements with them.” Participants also encouraged digital peer support specialists to be mindful of digital fatigue while setting boundaries with service users. A participant stated, “just being mindful of when you are starting to get that fatigue and being able to go, ‘okay, I need to take a break.’”

### Monitoring Digital Peer Support and Addressing Digital Crisis

The eighth theme, monitoring digital peer support and addressing digital crisis, constituted 8.2% (12/146) of the themes discussed in the focus groups. Transparency about the role of a digital peer support specialist was encouraged while addressing digital crisis:

Understanding one’s role as a peer support specialist, versus a different kind of service provider. A peer specialist is not a person who is geared or trained to respond to a full-fledged crisis. By nature, most peer specialists, unless they’re in an emergency response setting, are not trained or oriented to respond to crisis situation.

However, multiple participants encouraged the formation of trainings around digital crisis intervention. For example, one participant said, “if not a training, even just a conversation about how to respond to someone that is in crisis virtually...so I think that’s a really important discussion that needs to be had and maybe training that needs to be developed.” Peer support specialists questioned how they could better support service users, virtually, while in a crisis. A participant stated, “It’s very easy to sit with someone and make them feel not alone, but how can we make them feel not alone, when they can click a button and cut us off?”

### Peer Support Competencies

The ninth theme consisted of peer support competencies and included the subtheme self-determination. The theme of peer support competencies constituted 5.5% (8/146) of the themes discussed in the focus groups. The majority of participants believed that peer support competencies were essential to digital success. A participant stated, “I think still an essential ability is really the fundamentals of peer support.” Empathy, engagement, mutuality, sharing lived experiences, and the cultivation of hope were some of the competencies mentioned that peers believed were important to both in-person and digital peer support. For example, a participant said, “the ability to cultivate hope, to communicate, to partner with, to enhance self-determination, are all things that feel, to me, very important, as well as abilities.” The majority of participants believed that the peer support competencies developed by SAMHSA aligned with those of digital peer support. A participant stated, “relationship focused, trauma informed: all of this should definitely align with digital peer support.”

### Health Literacy

The last theme, health literacy, was an emerging theme that constituted 1.4% (2/146) of the themes discussed in the focus groups. Many participants emphasized the importance of health literacy in digital peer support. One participant encouraged the use of a “checklist” as a mechanism of checking “basic digital health literacy.” Communication with, and extracting information from, members of the medical field was cited as a challenge for peers. For example, one participant shared, “[COVID-19] has definitely made it hard to get information that I need from doctors to deal with my members...trying to get the doctors to connect with me at the same time is a work in progress.” Some participants believed more knowledge around digital peer support may help with peer-to-doctor communication during COVID-19. A participant stated, “I feel as if that with digital and the more knowledge we have and the more educational stuff we can put out with the doctors and also in our members, it will definitely help during this time at COVID.”

## Discussion

### Principal Findings

The purpose of this study was to introduce the coproduction of core competencies for digital peer support specialists to promote the consistency and standardization of best practices. The following themes emerged from the four focus groups with 59 participants: (1) protecting the rights of service users, (2) technical knowledge and skills in the practice of digital peer support, (3) available technologies, (4) equity of access, (5) digital communication skills, (6) performance-based training, (7) self-care, (8) monitoring digital peer support and addressing digital crisis, (9) peer support competencies, and (10) health literacy (emerging). The established competencies may act as a guide for training and skill development to be integrated into state peer support specialist competencies and enhanced established competencies endorsed by SAMHSA.

Peer support specialists are increasingly using mobile and online technologies to deliver peer support [19]. While SAMHSA has developed core competencies for in-person peer support, peer support’s rapid transition to digital forms during the COVID-19 pandemic requires the expansion and redefinition of skills and abilities for peer support specialists who are now offering services and support online and through digital platforms. For example, participants in the focus groups recommended that digital peer support specialists require knowledge about digital platforms and devices and on cybersecurity and privacy. Digital peer support specialists need specific skills and abilities to communicate effectively with service users via technology while still maintaining boundaries between work and personal life. Based on the themes that emerged from the focus groups, the authors make a number of recommendations, as discussed in the following sections.

### Ensure Equity in Digital Peer Support Delivery

Technological advances can lead to health care disparities for underresourced populations. It is important for those who develop digital health technologies to understand the challenges faced by disadvantaged populations, including older adults; people who have mobility or cognitive challenges; lesbian, gay, bisexual, and transgender people; justice-involved people; and Native American groups. Interventions designed with community-engagement methods may be better equipped to address inequities, health disparities, and linguistic and cultural considerations. The decision to *meet people where they are* individually, culturally, and digitally can benefit the implementation of digital interventions and help to achieve health equity [27]. Digital peer support specialists need to be aware of mental health service users’ biopsychosocial needs and leverage technology tools that can enhance service and resource delivery. For example, digital peer support specialists should communicate using large font when communicating with older adults due to common impairments in vision and should include closed captioning while communicating with adults who are hard of hearing. Digital communication skills also include peer support specialists’ ability to adapt their background setting to the needs of the service user. Digital peer support specialists should communicate in a well-lit setting with low background noises to ensure that service users can correspond comfortably.

### Understand Available Technologies and Analytical Techniques

Technology is frequently changing. Being fluent and up to date in technology literacy may facilitate a peer support specialists’ capacity to educate service users on the dynamics of digital technologies. Research, to date, indicates that peer support specialists and service users are commonly not informed of benefits and disadvantages of using specific technologies and their respective analytical techniques. Thus, some technologies and respective analytical strategies may not be aligned with peer support specialists’ values and practice principles [17]. For example, research indicates that individuals, including peer support specialists and service users that have a unique social-cultural history with the mental health system, may not perceive a chatbot as ethical. Chatbots constitute a software that allows for online text with an artificial (ie, nonhuman) agent. With mastery of technology literacy, digital peer support specialists could adhere to the technological preferences (eg, if they prefer a certain digital platform) and accessibility (eg, if service users do not have financial access to certain digital devices) of service users. Peer support specialists would have the skill to use clear, understandable, and value- and judgement-free language, and would have the tools to discuss technologies and to support themselves and service users in making informed decisions in engaging with technologies.

### Preventing Digital Fatigue Through Separating Work and Personal Life

Unlike in-person peer support, digital peer support can be offered anytime and anywhere. As a result, digital peer support may lead to more frequent and more casual interactions and behaviors. Separating work from personal life is important for avoiding digital fatigue. “Digital fatigue” is defined as a state of mental exhaustion and reduced motivation and concentration due to the overuse of digital devices and platforms [28]. Digitalization of the workplace has been cited as a common reason for a decrease in energy levels [28]. During COVID-19, the majority of business activities have been performed through digital platforms [29]. Separating work and personal life is also important for avoiding potential boundary issues that could occur in asynchronous communications. For example, providing feedback during nonbusiness hours could lead service users to expect nearly instantaneous feedback. Service users’ unimpeded access to digital peer support specialists could potentially lead to digital fatigue, exhaustion, and increased burnout rates. Similar to other fields of study, it is important for digital peer support specialists to maintain normal hours and locations, ensure timely and consistent feedback, and ensure private, consistent, and professional meetings (eg, do not smoke cigarettes during a meeting). A supportive management structure may be important for facilitating self-care. A supportive management structure offers ongoing support, is nonpunitive, and has been shown to be effective with peer support specialists [1].

### Self-Determination Is Key to Engagement

Supporting services users’ preferences for available technologies has the potential to promote engagement in digital peer support services. Self-determination theory represents a general theory of human motivation that explicitly identifies autonomy as a human need that, when supported, facilitates more autonomous forms of motivation [30]. Promoting service users’ autonomy, then, might promote better mental and physical health management. Service users with greater autonomy orientations might also be more motivated to make positive health-related behavior changes [30]. Shared decision making in research allows for greater personal autonomy and takes into account the extent to which service users want to be involved in the decision-making process [31]. Shared decision making in research proposes that individual preferences—what matters to service users and families—should play a major role in determining health care decisions [31]. The majority of peer support services are offered through videoconference-based services, smartphone services, and social media, followed by burgeoning services offered through virtual reality and video games. Peer support organizations are developing multiple ways for people to connect based on preference.

### Protecting the Rights of Service Users

Complying with privacy laws, such as the Health Insurance Portability and Accountability Act (HIPAA) Privacy Rule, protects individuals’ personal health information and sets boundaries on the use and release of health records [32]. It establishes the appropriate safeguards that health care providers and others must adhere to in order to protect the privacy of health information, and it sets standards for HIPAA-compliant software and products [32]. Communication through, and storing information in, HIPAA-compliant devices ensures the protection of electronic health information [33]. Storing information, records, and devices in a secure location and using trusted networks increases cybersecurity measures and helps provide a private, consistent, and professional setting [33]. International privacy laws include Australia’s Privacy Law, the European Union’s General Data Protection Regulation, Canada’s Personal Information Protection and Electronic Documents Act, and Singapore’s Personal Data Protection Act. Like HIPAA, these privacy laws safeguard personal data and provide guidelines on security storage and informed consent [34]. Understanding cybersecurity and how to secure one’s phone, laptop, remote desktop, and data storage is important to digital peer support specialists’ ability to ensure privacy and confidentiality for their service users. Understanding where information is being stored, for example, on iCloud, is also important to digital peer support specialists’ ability to protect service users’ data. Digital peer support specialists can potentially increase cybersecurity measures by securing all devices, checking that VPNs (virtual private networks) and firewalls have necessary updates, uploading the most recent security patches, using strong passwords, implementing multifactor authentication, and only accessing trusted networks or cloud services—not free hotspots [35]. Digital peer support specialists are required to obtain informed consent from service users and to conduct meetings in a confidential setting. Digital peer support specialists must keep information discussed confidential, yet mandated reporting is required when abuse is observed or suspected. Digital peer support specialists need the capacity to inform service users of their rights and of what data are collected by technologies at the time of service. For example, messaging on Facebook Messenger falls under the category of “nonpublic-facing” communication in the US Office for Civil Rights, meaning that only intended parties are allowed to participate in communication. However, health information provided on Facebook’s chat platform is unprotected under HIPAA [36]. Health information provided through Facebook may be regulated under Facebook’s privacy policy, but it is not subject to HIPAA standards [36].

### Technical Knowledge and Skills in the Practice of Digital Peer Support

Lastly, as an overarching recommendation, peer support specialists are encouraged to ensure competence in the digital peer support services they provide. This includes acquiring the skills and knowledge necessary to comfortably use the technologies being used. For example, digital peer support specialists should have the ability to navigate videoconferencing software and have basic knowledge on devices like computers and tablets. Technical knowledge and skills could potentially equip digital peer support specialists with the ability adapt to technical difficulties, such as communicating with service users who have a poor data signal. At a minimum, this should include consultation with information technology professionals, web design developers, and expert digital peer support specialists in the field. Practicing with different digital platforms or devices and seeking feedback is important for skill development. However, having advanced expertise in technology skills and troubleshooting may not be the role of peer support specialists. As such, having a dedicated person on-site, in person, or virtually, who understands the philosophy, values, and practices of peer support specialists, could facilitate advanced troubleshooting. That individual could be a peer or a nonpeer. Ongoing consultation with experts in the field of digital peer support will be important for peer support specialists who are new to digital peer support. All peer support specialists are encouraged to seek continuing education and ongoing consultation from their supervisors and others regarding any work and personal life issues that arise at any point. In Table 3, we outline recommendations for digital peer support competencies and provide some examples of support efforts.

**Table 3:** Authors’ recommendations for digital peer support competencies based on focus group analysis.

Authors’ recommendations for digital peer support competencies	Examples of digital peer support efforts to enhance best practices
Ensure equity in digital peer support delivery	Interventions should be designed using community-engagement methods to address inequities, health disparities, and linguistic and cultural considerations.
Understand available technologies and analytical techniques	Gaining technology literacy is important to digital peer support specialists’ ability to adhere to and use technologies that best fit the preferences and accessibility of service users.
Prevent digital fatigue through separating work and personal life	Digital peer support specialists should maintain normal hours and locations and ensure timely and consistent feedback to maintain boundaries and prevent exhaustion.
Self-determination is key to engagement	Supporting service users’ technology preferences has the potential to promote engagement in digital peer support services.
Protect the rights of service users	Complying with privacy laws, such as HIPAA^a^, can help protect service users’ personal health information and ensure the secure storage of records and safe communication.
Technical knowledge and skills in the practice of digital peer support	Practicing with different digital platforms or devices and seeking feedback on skill development can help digital peer support specialists to acquire the skills necessary to comfortably use technologies used in digital peer support.

^a^HIPAA: Health Insurance Portability and Accountability Act.

### Limitations

This study is not without limitations. First, there are potentially other competencies that have not been identified. Second, the sample lacked diversity based on racial and ethnic background and age. Future studies should consider the inclusion of disadvantaged populations, such as Hispanic, Latinx, and LGBTQIA (lesbian, gay, bisexual, transgender, queer/questioning, intersex, and asexual/aromantic/agender) populations. Third, as technology changes, so will these competencies. Fourth, the findings cannot be generalized to all digital peer support specialists, as the participants were selected from one listserv, which may have biased the results. In addition, it is unknown if there was international saturation. However, as the purpose of this study was to introduce the development of digital peer support competencies, future research could consider saturation of qualitative inquiry with an international sample. Fifth, the data could not be stratified by the role of the participant, and the data could not be stratified by volunteer-run services versus paid professional services. Sixth, the percentage of participants that completed a training in digital peer support is unknown and could have potentially biased the data. Seventh, the interview guide was not pretested, as it was developed with end users as partners. Further, we used an iterative process to modify the guide based on the focus groups. Eighth, focus group participants were not asked whether they were paid or volunteer peer support specialists, which may have introduced variation in the themes. Ninth, peer support has been around in some form for centuries. Note, the interest from the research community is more recent (ie, beginning with the seminal paper by Solomon [2]), and the interest from the health care field is very recent. Thus, some instances of cited references may not be referring to the more current use of peer support. Last, there may be certain functions of peer support that are not appropriate for technology. As such, exploring digital formats versus nondigital formats could explain which technologies are well suited for individuals. Future research looking at the integration of digital peer support competencies within digital peer support supervision is needed.

### Conclusions

Introducing the definition of digital peer support competencies is the first step to creating a standardization of best practices. Core competencies identify the skills and abilities that all digital peer support specialists need in order to provide support to people recovering from a mental health or substance use condition. These competencies may create consistency among digital peer support programs and could be used to develop potential reimbursement models. The COVID-19 pandemic has resulted in a rapid expansion of digital peer support services. The identification and definition of abilities that peer support specialists will need in order to offer services and support online and through digital platforms will be used to guide the training and practice of digital peer support. The core competencies listed establish how skills and values can be applied and verified through practice. Future research should work to verify and build off of the digital peer support competencies defined in this study.

As this study is the initial first step toward the development of digital peer support competencies, these competencies can be used as a starting point for organizations (eg, hospitals and community mental health centers) and businesses (eg, peer-led or coproduced telehealth commercial start-ups). The authors recommend the collective review of these competencies with peer support specialists, patients, caregivers, family members of people with mental health challenges, clinicians, administrators, and policy makers to determine the value of integrating each competency into their system of care. Policy makers may consider collective review and determination of the value of the inclusion of these initial competencies as part of (1) private and public health insurance, (2) peer support accreditation standards (eg, CARF [Commission on Accreditation of Rehabilitation Facilities] International and the Joint Commission), or (3) enhancements to SAMHSA’s existing competencies. As geographic regions and needs of systems of care and service users differ, organizations and businesses can select a milieu of competencies as a starting point and consider the integration of each competency based on their specific needs.

## Abbreviations

ACT: Assertive Community Treatment
CARF: Commission on Accreditation of Rehabilitation Facilities
HIPAA: Health Insurance Portability and Accountability Act
LGBTQIA: lesbian, gay, bisexual, transgender, queer/questioning, intersex, and asexual/aromantic/agender
RADaR: rigorous and accelerated data reduction
SAMHSA: Substance Abuse and Mental Health Services Administration
VPN: virtual private network
